# The Need for Glaucoma Management in Glaucoma Patients with Concurrent Obstructive Sleep Apnea: A Population-Based Cohort Study

**DOI:** 10.3390/biomedicines11010187

**Published:** 2023-01-11

**Authors:** Chia-Yi Lee, Hsiang-Wen Chien, Jing-Yang Huang, Chieh-Hung Yen, Hung-Chi Chen, Yih-Shiou Hwang, Chao-Kai Chang, Shun-Fa Yang

**Affiliations:** 1 Institute of Medicine, Chung Shan Medical University, Taichung 40201, Taiwan; 2 Nobel Eye Institute, Taipei 100008, Taiwan; 3 Department of Ophthalmology, Jen-Ai Hospital Dali Branch, Taichung 41265, Taiwan; 4 Department of Ophthalmology, Cathay General Hospital, Taipei 106438, Taiwan; 5 Departments of Ophthalmology, Sijhih Cathay General Hospital, New Taipei City 221037, Taiwan; 6 School of Medicine, College of Medicine, Fu Jen Catholic University, New Taipei 242062, Taiwan; 7 School of Medicine, National Tsing Hua University, Hsinchu 300044, Taiwan; 8 Department of Medical Research, Chung Shan Medical University Hospital, Taichung 40201, Taiwan; 9 Department of Ophthalmology, Chang Gung Memorial Hospital at Linkou, Taoyuan City 333423, Taiwan; 10 Graduate Institute of Biomedical Engineering, Chang Gung University, Taoyuan 33302, Taiwan; 11 Department of Medicine, Chang Gung University College of Medicine, Taoyuan 33302, Taiwan; 12 Center for Tissue Engineering, Chang Gung Memorial Hospital, Linkou 333423, Taiwan; 13 Department of Optometry, Da-Yeh University, Chunghua 51500, Taiwan

**Keywords:** glaucoma, obstructive sleep apnea, treatment, population-based, surgery

## Abstract

We try to evaluate glaucoma management numbers in patients with both glaucoma and obstructive sleep apnea (OSA) using the National Health Insurance Research Database (NHIRD) of Taiwan. A retrospective cohort study was conducted and patients with glaucoma were enrolled and divided into the OSA and non-OSA populations. A total of 11,778 participants were selected in both the OSA and non-OSA groups. The primary outcomes were the number of anti-glaucomatous medications each year and the total number of glaucoma laser and glaucoma surgeries. The Cox proportional hazard regression was utilized to produce the adjusted hazard ratios (AHR) with corresponding 95% confidence intervals (CI) between the two groups. After a study period of 18 years, 286 and 352 events of laser and surgeries for glaucoma were found in the OSA and non-OSA groups, respectively. After considering the effect of potential confounders, no significant difference concerning the numbers of laser trabeculoplasty, trabeculectomy and tube shunt surgery, cyclodestructive procedure and eyeball removal were found between the two groups (all 95% CIs included one). In addition, the multiple anti-glaucomatous medication usages were similar between the two groups (all *p* > 0.05) In the subgroup analyses, glaucoma patients older than 60 years and with OSA received significantly lesser trabeculectomy and tube shunt surgery compared to glaucoma patients older than 60 years without OSA (AHR: 0.774, 95% CI: 0.611–0.981) while other analyses revealed insignificant results (all 95% CIs included one). In conclusion, the presence of OSA does not increase the need for glaucoma management.

## 1. Introduction

Glaucoma is a prevalent ocular disease that causes large numbers of visual impairments and legal blindness worldwide [1,2]. According to an epidemiological study, the proportions of preceding undetected glaucoma were highest in the population living in the African and Asia regions [3]. Glaucoma can be divided into different categories including open-angle glaucoma (OAG), angle-closure glaucoma (ACG) and normal tension glaucoma (NTG) with different pathophysiology and clinical features [4,5]. The anti-glaucomatous medications, laser therapy and surgery have been used to manage glaucoma [1], while the retinal nerve fiber layer (RNFL) damage in glaucoma cannot be recovered despite proper treatment [4].

Regarding the risk factors of glaucoma, old age is a well-established predisposing factor for glaucoma, and individuals aged older than 40 years are under a higher risk of glaucoma development and the prevalence of glaucoma reached 3.3% percent in those aged more than 70 years old [6,7,8]. Refractive error is also a prominent risk factor for glaucoma in which myopia was related to OAG development while hyperopia was associated with ACG [8,9]. Other risk factors of glaucoma include crowded ocular structure, female sex, diabetes mellitus (DM), thin cornea and family history of glaucoma [6,7]. Recently, ischemia of the retina and optic nerve head has been proposed as a predisposing factor of glaucoma but without firm conclusions [10].

Obstructive sleep apnea (OSA) is a disease resulting from impaired ventilation [11], which can lead to a surge of sympathetic tone, hypercapnia, hypoxia and unstable blood flow [12,13,14]. Although there are some conflicting results [15], previous literature has demonstrated the possible relationship between OSA and glaucoma [16]. Because OSA can lead to ischemia and hypoxia and both conditions contribute to the RNFL damage [17,18,19], patients with glaucoma and OSA may experience a more recalcitrant course of glaucoma, which has been demonstrated in previous studies [20,21,22]. Still, whether the treatments for glaucoma would increase more in the OSA population than in the general glaucoma population needs further investigation.

The purpose of the current study is to survey the management numbers of glaucoma between OSA and non-OSA patients using the National Health Insurance Research Database (NHIRD) of Taiwan. The management of glaucoma is based on the number of anti-glaucomatous medications, arrangement of laser treatment and performance of glaucoma surgery.

## 2. Materials and Methods

### 2.1. Data Source

The NHIRD contains claims data of the National Health Insurance of nearly all inhabitants in Taiwan between 1 January 2000 and 31 December 2018. Our study obtained these data from the Longitudinal Health Insurance Database 2005 (LHID 2005), which is a sub-database of NHIRD and contains the records of nearly 2 million patients, which were randomly sampled from the NHIRD registry in the year 2005, and their data were connected from 2000 to 2018. Both the Ninth and Tenth Revisions of International Classification of Diseases were used for disease diagnosis in the NHIRD/LHID 2005. The accuracy of diagnosis is dependent on the physicians who enter the codes, and the system also provides the demography, time of medical visit, department codes, insurance codes of examination and codes of medications.

### 2.2. Patient Selection

**Figure 1:**
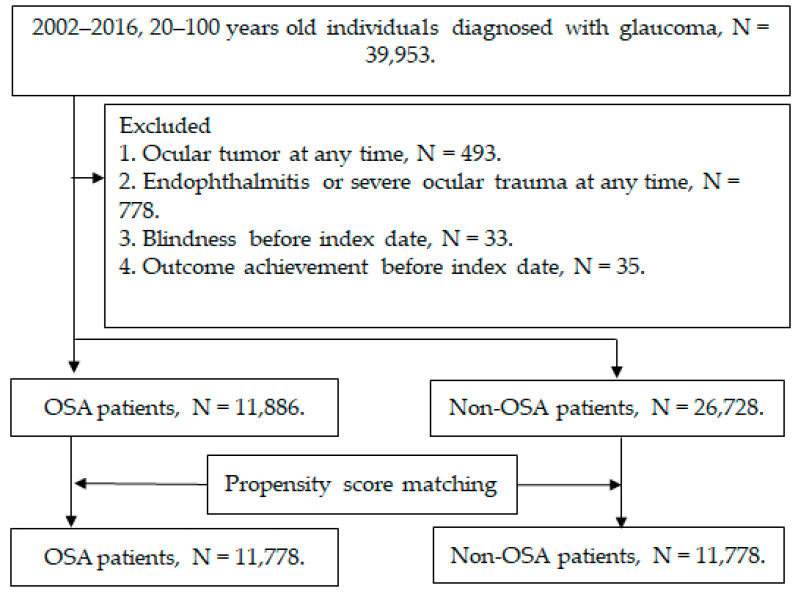
The flow chart of subject selection. N: number, OSA: obstructive sleep apnea.

We defined individuals as having glaucoma if their medical documents revealed (1) the presence of a glaucoma diagnosis; (2) the arrangement of intraocular pressure (IOP) measurement before glaucoma diagnosis; (3) the arrangement of visual field test, optical coherence tomography or fundoscopy before or on the same day of glaucoma diagnosis; and (4) the receipt of glaucoma diagnosis in ophthalmic outpatient department (OPD) and received treatment by an ophthalmologist. The index date was the date that the glaucoma diagnosis was entered into the NHIRD/LHID 2005. The exclusion criteria in this study were illustrated as follows: (1) receipt of legal blindness diagnosis before the index date; (2) receipt of an ocular tumors diagnosis at any time; (3) receipt of endophthalmitis or severe ocular trauma diagnoses at any time; and (4) outcome develops before the index date. Then, the patients with OSA were selected from the whole glaucoma population based on these criteria: (1) the presence of an OSA diagnosis, (2) the arrangement of polysomnography before the OSA diagnosis, and (3) the OSA diagnosis was entered by a thoracologist, otorhinolaryngologist or neurologist. After that, each individual with both OSA and glaucoma was matched to one glaucoma patient without OSA via the propensity score-matching (PSM) method according to demographic data, co-morbidities and medications listed in the following sections, and the non-OSA population constructed the control group. Subjects in the OSA group that could not be matched to one non-OSA patient were excluded from this study. A total of 11,778 patients were enrolled in both the OSA and non-OSA groups after the selection process. In addition, participants in the study population were categorized into OAG, NTG and ACG subgroups according to the diagnostic codes and differences in outcomes of these subgroups between OSA and non-OSA populations were analyzed. The flow chart of subject selection is revealed in Figure 1.

### 2.3. Primary Outcome Measurement

The performance of glaucoma treatments, which were the primary outcome of our study, was defined as the following: (1) the number of topical anti-glaucomatous medications used, which included beta-blockers, alpha agonists, carbonic anhydrase inhibitors, prostaglandin, and miotic agents; (2) the arrangement of laser trabeculoplasty; (3) the arrangement of trabeculectomy and tube shunt surgery; (4) the arrangement of cyclodestructive procedure; and (5) the arrangement of eyeball removal. To prevent the overestimation of the primary outcome, the primary outcome was considered to develop only in patients who received introductory diagnostic codes, medical prescriptions and surgical treatments from an ophthalmologist.

### 2.4. Demographic Variables and Comorbidities

To make secure that the health status and the potential risk factors of advanced glaucoma between the OSA group and non-OSA group are sufficiently comparable, we investigated the influence of enrollment years, age, sex, urbanization, education level, and the following morbidities and medications in the multivariate model: hypertension, DM, ischemic heart diseases, hemodialysis, cerebrovascular disease, uveitis, cataract, refractive error, and steroid application including topical and systemic prednisolone, methylprednisolone, hydrocortisone, triamcinolone and dexamethasone. We longitudinally followed the data from the index date until the date of each outcome achievement, subject withdrawal from the National Health Insurance program, or till the end date of NHIRD/LHID 2005, which is 31 December 2018.

### 2.5. Statistical Analysis

The SAS version 9.4 (SAS Institute Inc., Cary, NC, USA) was applied for all the analyses. After PSM of the OSA and non-OSA groups at a 1:1 ratio, descriptive analyses were used to show the difference of basic characters between the two groups, and the absolute standardized difference (ASD) between the two groups was also calculated and an ASD < 0.1 was regarded as a similar value. Then, the incidence rate and the adjusted hazard ratios (AHR) with corresponding 95% confidence intervals (CI) of primary laser and surgical outcome between the two groups were yielded through Poisson regression and Cox proportional hazard regression. The Cox proportional hazard regression incorporated the demographic data, systemic comorbidities, ocular comorbidities and steroid utilization. In the subgroup analyses, we stratified the OSA and non-OSA groups via age, gender, glaucoma subtypes (i.e., OAG, ACG and NTG), the duration of OSA, and the presence of OSA-related surgery (i.e., the uvulopalatopharyngoplasty, tonsillectomy, pharyngoplasty, maxillary mandibular advancement, septoplasty, turbinoplasty, nasal valve surgery, adenoidectomy and tongue body reduction). We then compared the incidence of laser trabeculoplasty, trabeculectomy and tube shunt surgery, and cyclodestructive procedure via the Cox proportional hazard regression again. Of note, the eyeball removal was not compared at this stage due to insufficient patient numbers. We produced the Kaplan–Meier curve to illustrate the cumulative incidence of laser and surgical management between the two groups, and used log-rank test to determine the significance between two survival curves. For the anti-glaucoma medications, the percentage of any anti-glaucomatous medication usage and the percentage of more than one type of anti-glaucomatous medication applications between the two groups in every year was analyzed via independent T test and presented as a line chart. The statistical significance was regarded as *p* < 0.05, and *p* values less than 0.0001 were presented as *p* < 0.0001.

## 3. Results

The baseline characteristics between the OSA and non-OSA groups are shown in Table 1. The years of the index date, age and gender were nearly identical between the two groups due to the PSM process (all ASD < 0.100). In addition, other demographic data, co-morbidities and steroid application were also similar between the OSA and non-OSA groups (all ASD < 0.100). Still, the application of prednisolone was significantly higher in the OSA group compared to the non-OSA group (19.89% versus 15.66%, ASD = 0.1110) (Table 1).

**Figure 2:**
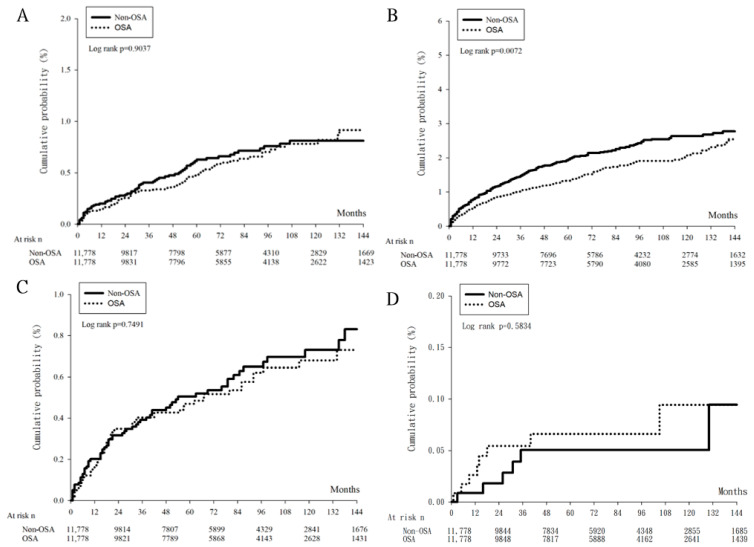
The cumulative probability of glaucoma management between obstructive sleep apnea group and control group. N: number, OSA: obstructive sleep apnea. (**A**) The cumulative probability of laser trabeculoplasty between the two groups, (**B**) The cumulative probability of trabeculectomy and tube shunt surgery between the two groups, (**C**) The cumulative probability of cyclodestructive procedure between the two groups, (**D**) The cumulative probability of eyeball removal between the two groups.

**Figure 3:**
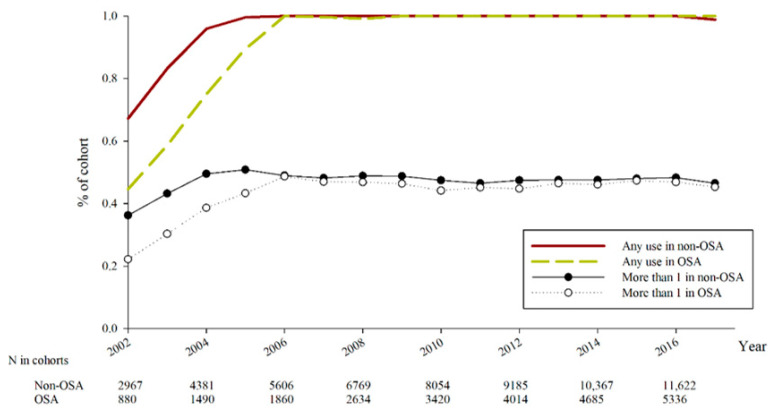
The number of anti-glaucomatous medications in every year between obstructive sleep apnea group and control group. N: number, OSA: obstructive sleep apnea.

After a study period of 18 years, there were 286 and 352 events of laser and surgical management for glaucoma in the OSA and non-OSA groups, respectively (Table 2). Considering the effect of potential confounders, no significant difference concerning the incidence of laser trabeculoplasty, trabeculectomy and tube shunt surgery, cyclodestructive procedure and eyeball removal between the OSA and non-OSA groups were observed (all the 95% CIs included one) (Table 2). The cumulative probabilities of the four outcomes between the two groups are demonstrated in Figure 2 and the log-rank test also revealed insignificant differences (all *p* > 0.05). Regarding the numbers of anti-glaucomatous medications, both the percentage of any anti-glaucomatous medication usage and the percentage of multiple anti-glaucomatous medications application showed similar values between the OSA and non-OSA groups (all *p* > 0.05) (Figure 3).

In the subgroup analyses, the glaucoma patients aged more than 60 years and with OSA received significantly fewer trabeculectomy and tube shunt surgery compared to the glaucoma patients aged more than 60 years without OSA (AHR: 0.774, 95% CI: 0.611–0.981). However, the incidence of glaucoma laser and surgical managements demonstrated similar values in all other subgroups stratified via age, gender, glaucoma subtype including the OAG, NTG and ACG, OSA duration, or presence of OSA-related surgeries (all the 95% CIs included one) (Table 3).

## 4. Discussion

In our study, we found that co-existing OSA in glaucoma patients does not increase the incidence of advanced glaucoma management such as laser treatment and glaucoma surgery. Moreover, the percentage of patients with more than one anti-glaucomatous medication usage is similar between the OSA and non-OSA populations. On the other hand, the ratio of trabeculectomy and tube shunt surgery is lower in those glaucoma individuals with OSA and aged more than 60 years old.

The association between OSA and glaucoma has been evaluated for decades [15]. Generally, evidence has shown that OSA can elevate the incidence of glaucoma [7,23]. In a previous article, the odds ratio for the correction between OSA and glaucoma was significantly higher [5]. Other research also demonstrated that OAG and NTG patients had a higher apnea hypopnea index value [16]. Moreover, in a previous population-based database study conducted in the Taiwanese population, a higher incidence of OAG was found in those patients with OSA than in the non-OSA group [24]. Regarding the possible etiology of the relationship between OSA and glaucoma, OSA-induced hypoxia may cause the death of RNFL and follow glaucomatous neuropathy [6,17,19]. Moreover, the presence of OSA has been proven to trigger hypertensive and ischemic conditions in the human body [13,18,25]; thus, the impaired peripapillary vasculature and ischemia in such condition may contribute to the damage to the optic nerve head and the subsequent glaucoma development [6,10]. On the other hand, some research did not agree that there is an association between OSA and glaucoma: an earlier article illustrated the inconclusive relationship between OSA and glaucoma, and that the severity of OSA in the glaucoma population did not increase compared to those patients without glaucoma [26]. In addition, research has revealed the insignificantly higher glaucoma prevalence in OSA individuals after adjusting multiple potential risk factors of glaucoma [27]. Moreover, nasal steroids have been used in certain populations with OSA and the steroid itself can trigger the development of glaucoma [12,28,29]; thus, the effect of steroids on glaucoma may be mistaken as the effect of OSA. Overall, OSA may serve as a predisposing factor for glaucoma development, but further studies are needed to defend this concept. Since OSA may lead to glaucoma occurrence, we wonder whether the existence of OSA may not only lead to glaucoma development but also increase the corresponding management of glaucoma. The results of this study indicate that the possibility of such an association is relatively low.

The current study illustrated that the percentage of multiple anti-glaucomatous medication applications, the incidence of glaucoma laser therapy, and the ratio of glaucoma-related surgeries are similar between the OSA and non-OSA groups after adjusting multiple potential risk factors including steroids. To our knowledge, few studies have surveyed this issue concerning glaucoma severity and concurrent OSA before. Although some studies have proposed that glaucoma severity was associated with OSA [20,21,22], the little case numbers and the absence of categorizing glaucoma treatments limited the confidence of that research. The percentage of more than one anti-glaucomatous medication was approximately 40 percent in both the OSA and non-OSA groups, and the sum of glaucoma laser and surgery ever arranged was about 3 percent. Because of the emergence of fixed combination anti-glaucomatous medicines, the number of anti-glaucomatous medications needed to control glaucoma progression has decreased nowadays compared to previous periods, and patient compliance has increased [30]. Consequently, the necessity of multiple anti-glaucomatous medications to control glaucoma these days indicates a more complicated course. The existence of OSA did not trigger a higher ratio of glaucoma medication requirements compared to non-OSA glaucoma patients, which may imply that the influence of OSA on glaucoma, if present, is not strong enough to aggregate the glaucoma progression. Likewise, laser treatment and glaucoma surgery are preserved for those with severe glaucoma due to advancements in anti-glaucoma medications and the lower quality of life for those having glaucoma surgery [30,31]. Both the low incidence of glaucoma laser as well as surgery, and the insignificant difference between the two groups, indicate the minimal effect of OSA on glaucoma progression and severity. Interestingly, the incidence of multiple anti-glaucomatous medications, laser treatment and glaucoma-related surgeries was numerically lower in the OSA group than that in the non-OSA group. We speculate that the OSA itself is correlated to several ocular disorders such as floppy eyelid syndrome, dry eye disease, diabetic retinopathy and hypertensive retinopathy, which lead to ocular irritation or impaired vision [5,32,33]. Consequently, these patients may come to ophthalmic OPD for medical assistance and only the underlying early glaucoma may be found; thus, only simple management is warranted for them.

In the subgroup analyses of this study, the impact of OSA on glaucoma management did not alter with different conditions. The only significant finding is the lower incidence of trabeculectomy and tube shunt surgery in the OSA population older than 60 years old than in the non-OSA population with the same age interval. The possible explanation is that the OSA-induced ocular surface irritation and retinal diseases meant that the patients came to the ophthalmic department more frequently [5,32], and certain glaucoma was found before prominent progression. Age is a significant risk factor for OSA and glaucoma development [1,6,34]; thus, the prominent effect of age in those older than 60 years old may interfere with the effect of OSA on glaucoma severity. Still, the upper limit of the 95% CI for trabeculectomy and tube shunt surgery incidence in the OSA group was very close to one. Accordingly, we would not regard this finding as an important issue but only a marginal difference, which shows insignificant clinical relevance. On the contrary, the gender, types of glaucoma, duration of OSA and OSA-related surgery showed scant effects on glaucoma management. In previous reports, female gender was a risk factor for glaucoma [35], and they were often associated with the development of OAG and NTG [16]. The minimal difference in glaucoma surgery between different gender, glaucoma types and OSA duration further illustrated that OSA would not exaggerate the progression of glaucoma regardless of other predisposing factors. Of note, NTG is frequently associated with OSA in previous literature [36,37,38], but the result of our study implied that the number of treatments is not higher in the OSA patients with NTG compared to general NTG patients. We speculated that maybe the OSA-related NTG presents with more severe optic nerve damage, but the treatment remains similar due to the low IOP. In addition, the presence of OSA-related surgeries did not cause a higher number of glaucoma management. In Taiwan, since most OSA-related surgeries are arranged if the continuous positive airway pressure and weight-loss therapy showed a poor effect, the OSA severity might contribute little effect on glaucoma management, which needs further study.

Regarding the demography between the OSA and non-OSA groups, the majority of covariates revealed similar distribution between the two groups even though OSA was diagnosed more commonly in males and those with metabolic disorders [34,35,39]. The reason is that the PSM process considers each parameter between two matched individuals. The only parameter with a significantly different distribution between the two groups was the usage of oral or topical prednisolone. Because prednisolone can be used in various ophthalmic diseases and cause ocular hypertension [40], the higher ratio of prednisolone in the OSA group may hamper the results. Nevertheless, the other steroid applications between the two groups were similar; thus, the general steroid utilization ratio was nearly identical and might not influence the outcome evaluation. Moreover, all the parameters were enrolled in the multivariable analyses and their effects on glaucoma severity were considered and adjusted. Consequently, the higher ratio of prednisolone application did not contribute to significantly influence this study.

There are several limitations presented in the current study. Firstly, the claimed database nature of our data source meant that many important indexes could not be accessed, which included the polysomnography results throughout the OSA management, the application of continuous positive airway pressure device, the arrangement of weight-loss therapy, the details of the OSA treatment course, the results of glaucoma-related examination including the visual field optical coherence tomography, the fluctuating of IOP after treatment in both the OSA and non-OSA groups, the controlling of IOP and glaucoma damage progression after glaucoma management, and the exact severity of glaucoma and other ocular diseases. Because the IOP value was unknown in the NHIRD/LHID 2005 and the main mechanism of OSA-related glaucoma could be hypoxia and ischemia [6,13,19], we could not ensure that glaucoma in the OSA group had a similar severity to the non-OSA group or the hypoxia and ischemia-induced glaucoma in a lower IOP status; thus, no additional surgical treatment may be warranted in the OSA group despite advanced disease. Accordingly, we can only access the number of glaucoma interventions rather than the actual glaucoma progression and optic nerve damage. In addition, a considerable number of patients were diagnosed with “unspecific glaucoma” and were excluded from the subgroup analyses concerning the glaucoma subtype, which may cause statistical bias. In addition, the design of the database stops tracking if any of the outcomes occurred; thus, we did not know whether a person with laser trabeculoplasty arrangement further received trabeculectomy in the following few months/years. Finally, because we excluded OSA patients who could not be matched to non-OSA individuals via PSM, about 108 participants were excluded and this may lower the statistical power. Still, both groups contained a patient number of more than 10 thousand after the selection process. Consequently, the influence of PSM on statistical power might not be significant.

## 5. Conclusions

In conclusion, the presence of OSA does not affect the management numbers of glaucoma concerning anti-glaucomatous medications, laser treatments and glaucoma-related surgeries. Furthermore, the insignificant effect of OSA on glaucoma management is universal in different types of glaucoma including OAG, NTG and ACG. Consequently, the therapeutic program of newly diagnosed glaucoma should not be altered in those with OSA. A further large-scale prospective study to evaluate the effect of continuous positive airway pressure devices on the severity and management of glaucoma in OSA individuals is mandatory.

## Figures and Tables

**Table 1:** Baseline characteristics among propensity score-matched population.

Characteristics	Non-OSA N = 11,778	OSA N = 11,778	ASD
Year of index			0.0000
2001–2006	2820 (23.94%)	2781 (23.61%)	
2007–2011	3858 (32.76%)	3917 (33.26%)	
2012–2017	5100 (43.30%)	5080 (43.13%)	
Age at index			0.0000
<40	612 (5.20%)	637 (5.41%)	
40–49	1172 (9.95%)	1160 (9.85%)	
50–59	2418 (20.53%)	2435 (20.67%)	
60–69	3348 (28.43%)	3310 (28.10%)	
70–79	2952 (25.06%)	2923 (24.82%)	
≧80	1276 (10.83%)	1313 (11.15%)	
Sex			0.0007
Male	4545 (38.59%)	4549 (38.62%)	
Female	7233 (61.41%)	7229 (61.38%)	
Urbanization			0.0376
Urban	7450 (63.25%)	7371 (62.58%)	
Sub-urban	3207 (27.23%)	3274 (27.80%)	
Rural	1121 (9.52%)	1133 (9.62%)	
Education level			0.0238
Elementary school or below	5783 (49.10%)	5894 (50.04%)	
Junior high school	1694 (14.38%)	1703 (14.46%)	
Senior high school	3268 (27.75%)	3177 (26.97%)	
University or above	1033 (8.77%)	1004 (8.52%)	
Co-morbidities			
Hypertension	5613 (47.66%)	5578 (47.36%)	0.0060
DM	3083 (26.18%)	3084 (26.18%)	0.0002
Ischemic heart diseases	1385 (11.76%)	1440 (12.23%)	0.0144
Hemodialysis	151 (1.28%)	154 (1.31%)	0.0023
Cerebrovascular disease	1173 (9.96%)	1240 (10.53%)	0.0188
Uveitis	402 (3.41%)	433 (3.68%)	0.0142
Cataract	5302 (45.02%)	5320 (45.17%)	0.0031
Refractive error	393 (3.34%)	487 (4.13%)	0.0421
Steroid use			
Prednisolone	1844 (15.66%)	2343 (19.89%)	0.1110
Methylprednisolone	563 (4.78%)	777 (6.60%)	0.0785
Hydrocortisone	264 (2.24%)	343 (2.91%)	0.0423
Triamcinolone	642 (5.45%)	879 (7.46%)	0.0819
Dexamethasone	1468 (12.46%)	1665 (14.14%)	0.0493
OSA-related surgeries		2537 (21.54%)	

OSA: obstructive sleep apnea, N: number, DM; diabetes mellitus, OAG: open-angle glaucoma, NTG: normal tension glaucoma, ACG: angle-closure glaucoma, ASD: absolute standardized difference.

**Table 2:** Incidence risk of study event among propensity score matched groups.

Event	Non-OSA n = 11,778	OSA n = 11,778
Laser trabeculoplasty		
Follow up person months	928,868	907,452
New case	69	67
Incidence rate ^#^(95% CI)	0.740 (0.590–0.940)	0.740 (0.580–0.940)
Crude relative risk (95% CI)	Reference	0.979 (0.700–1.371)
AHR (95% CI)	Reference	0.978 (0.698–1.371)
Trabeculectomy and tube shunt surgery		
Follow up person months	917,540	899,247
New case	239	182
Incidence rate ^#^(95% CI)	2.600 (2.290–2.960)	2.020 (1.750–2.340)
Crude relative risk (95% CI)	Reference	0.774 (0.655–0.914)
AHR (95% CI)	Reference	0.855 (0.720–1.016)
Cyclodestructive procedure		
Follow up person months	930,462	908,072
New case	64	60
Incidence rate^#^(95% CI)	0.690 (0.540–0.880)	0.660 (0.510–0.850)
Crude Relative risk (95% CI)	Reference	0.944 (0.664–1.343)
AHR (95% CI)	Reference	0.927 (0.651–1.320)
Eyeball removal		
Follow up person months	933754	910816
New case	6	8
Incidence rate ^#^(95% CI)	0.060 (0.030–0.140)	0.090 (0.040–0.180)
Crude Relative risk (95% CI)	Reference	1.343 (0.466–3.872)
AHR (95% CI)	Reference	1.284 (0.442–3.725)

OSA: obstructive sleep apnea, AHR: adjusted hazard ratio, CI: confidence interval. ^#^ Incidence rate, per 10,000 person-months.

**Table 3:** Stratified analysis for different parameters in glaucoma population.

Parameters	Laser Trabeculoplasty	Trabeculectomy and Tube Shunt Surgery	Cyclodestructive Procedure
Age			
Age < 60	0.899 (0.559–1.446)	0.966 (0.751–1.242)	0.836 (0.494–1.414)
Age ≥ 60	1.043 (0.720–1.511)	0.774 (0.611–0.998)*	0.856 (0.588–1.247)
Sex			
Male	1.070 (0.696–1.644)	0.833 (0.650–1.068)	0.721 (0.461–1.129)
Female	0.924 (0.622–1.374)	0.883 (0.694–1.125)	1.032 (0.671–1.588)
Glaucoma subtype			
OAG	1.066 (0.596–1.907)	1.114 (0.749–1.656)	0.970 (0.464–2.029)
NTG	7.095 (0.665–9.719)	1.180 (0.262–5.322)	NA
ACG	0.925 (0.532–1.609)	0.731 (0.514–1.038)	0.697 (0.381–1.274)
OSA years			
OSA < 1 year	1.489 (0.826–2.685)	1.322 (0.937–1.864)	0.502 (0.186–1.359)
OSA 1–2 years	1.413 (0.765–2.610)	0.977 (0.653–1.461)	0.659 (0.270–1.609)
OSA > 2 years	0.832 (0.590–1.173)	0.752 (0.614–1.022)	0.940 (0.677–1.305)
OSA-related surgeries			
With any surgery	1.047 (0.766–1.431)	0.824 (0.674–1.008)	0.829 (0.753–1.147)
Without surgery	0.724 (0.547–1.357)	0.883 (0.729–1.070)	0.899 (0.666–1.260)

OSA: Obstructive sleep apnea, N: Number, DM: Diabetes mellitus, OAG: Open-angle glaucoma, NTG: Normal tension glaucoma, ACG: Angle-closure glaucoma, NA: Not applicable. * denotes a significant difference between the obstructive sleep apnea and non-obstructive sleep apnea populations.

## Data Availability

Due to the policy of the National Health Insurance Administration in Taiwan, the raw data of this study are not available.
